# PDLIM2 is a novel E5 ubiquitin ligase enhancer that stabilizes ROC1 and recruits the ROC1-SCF ubiquitin ligase to ubiquitinate and degrade NF-κB RelA

**DOI:** 10.1186/s13578-024-01281-x

**Published:** 2024-07-30

**Authors:** Fan Sun, Gutian Xiao, Zhaoxia Qu

**Affiliations:** 1 Department of Microbiology and Molecular Genetics, UPMC Hillman Cancer Center, University of Pittsburgh School of Medicine, Pittsburgh, PA 15213 USA; 2 Department of Molecular Microbiology and Immunology, Hastings Center for Pulmonary Research, Norris Comprehensive Cancer Center, University of Southern California Keck School of Medicine, 1450 Biggy Street, Harlyne J. Norris Research Tower (NRT) 4506, Los Angeles, CA 90033 USA

**Keywords:** PDLIM2, NF-κB, RelA/p65, RBX1/ROC1, RBX1/ROC1 stabilization, SCF^β-TRCP^ E3 ubiquitin ligase, E3 ubiquitin ligase, Ubiquitination, Proteasomal degradation, E5 ubiquitin ligase enhancer

## Abstract

The PDZ-LIM domain-containing protein PDLIM2 is a common tumor suppressor and a key immune modulator. One main function of PDLIM2 is to promote the ubiquitination and proteasomal degradation of nuclear activated NF-κB RelA, a physiologically indispensable transcription factor whose persistent activation has been linked to almost all cancer types and inflammation-associated diseases. However, it remains unknown how PDLIM2 exerts this physiologically and pathogenically important function. Here, we show that PDLIM2 acts as a ubiquitin ligase enhancer, termed E5. It stabilizes ROC1, an essential component of SKP1/Cullin/F-box protein (SCF) ubiquitin ligases, and chaperones the ROC1-SCF^β-TrCP^ ubiquitin ligase to ubiquitinate nuclear RelA for proteasomal degradation in the nucleus. Consistently, silencing of ROC1, Cullin 1 or the F-box protein β-TrCP blocks RelA ubiquitination and degradation by PDLIM2. These data provide new mechanistic insights into how PDLIM2 promotes nuclear RelA ubiquitination and degradation, thereby serving as a critical tumor suppressor and a vital immune regulator. They also improve our understanding of the complex cascade of the ubiquitination and NF-κB pathways, particularly given the well-known role of the ROC1-SCF^β-TrCP^ ubiquitin ligase in initiating NF-κB activation by directly binding to and ubiquitinating NF-κB inhibitors for the proteasomal degradation in the cytoplasm.

**Supplementary Information:**

The online version contains supplementary material available at 10.1186/s13578-024-01281-x.

## Dear Editor

Under normal conditions, PDZ-LIM domain-containing protein 2 (PDLIM2, also known as Mystique and SLIM) is ubiquitously expressed, with the highest level in the lung [1]. At the cellular level, it is expressed strongly in lung epithelial cells and all major immune cell types [1–4]. Published data have linked PDLIM2 repression to a plethora of solid and liquid tumors, infectious diseases, and lung disorders [2–6]. Particularly, PDLIM2 is repressed in nearly all human lung cancer cases, and PDLIM2 repression is a causative driver of lung cancer, the leading cause of cancer death in both men and women [2, 3]. PDLIM2 repression also renders tumors resistant to the conventional chemotherapy as well as the innovative PD-L1/PD-1 immune checkpoint blockade therapy [2, 3]. Notably, systemic delivery of PDLIM2 by a clinically feasible nanotechnology (nanoPDLIM2) not only shows a promising therapeutic efficacy as a monotherapy in the mouse models of refractory lung cancer, but more importantly, in combination with PD-1 inhibitors and chemo drugs, completely eradicates all tumors in most animals without adding toxicity [3].

PDLIM2 exerts the indispensable tumor suppressor role mainly by promoting the ubiquitination and proteasomal degradation of nuclear activated RelA (also known as p65), thereby terminating the activation of this prototypic member of the nuclear factor-κB (NF-κB) family of transcription factors [2–5]. NF-κB plays critical roles in host defense and various physiological processes [7, 8]. Of note, persistent activation of NF-κB and RelA in particular, like PDLIM2 repression, has been linked to a legion of diseases and cancers, including lung cancer [7, 8].

Based on the structural similarity between the LIM (first identified in Lin-11, Isl-1 and Mec-3 and therefore named so) domain and the RING (really interesting new gene) finger domain, PDLIM2 had been proposed to act as a ubiquitin ligase [1]. RING finger proteins represent the largest family of ubiquitin ligases (E3s). Indeed, PDLIM2 became ubiquitinated in vitro in the presence of ubiquitin-activating enzyme (E1), ubiquitin-conjugating enzyme (E2) and ubiquitin, a general phenomenon seen with many RING finger proteins [1]. However, up to date, no LIM domain-containing proteins other than PDLIM2 have been reported to possess ubiquitin ligase activity. Moreover, it remains unknown if PDLIM2 functions by itself or forms a complex with other factors to promote protein ubiquitination and degradation. Most RING finger E3s consist of multiple subunits [9]. A typical RING finger E3 complex, such as the well-known SCF^β-TrCP^ ubiquitin ligase, contains the RING finger protein Regulator of Cullins-1 (ROC1, also known as Ring box protein-1, RBX1; RING finger protein 75, RNF75), the adaptor protein SKP1 (S-phase kinase-associated protein), the cullin protein Cullin 1 (CUL1), and the F-box protein β-TrCP (β-transducin repeat-containing protein; also known as F-box/WD repeat-containing protein 1A, FBXW1, FBXW1A or FWD1). In the complex, β-TrCP and ROC1 recognize protein substrates and recruit E2s, respectively. CUL1 is the scaffolding protein recruiting ROC1 and SKP1, and SKP1 links CUL1 to β-TrCP. One notable function of the SCF^β-TrCP^ ubiquitin ligase is to directly bind to and ubiquitinate NF-κB inhibitors for proteasomal degradation in response to NF-κB stimuli, unleashing RelA and other NF-κB members to enter the nucleus for gene transcription [7].

Here, we report that PDLIM2 promotes nuclear RelA ubiquitination and proteasomal degradation via the prototypic SCF^β-TrCP^ ubiquitin ligase. One essential function of PDLIM2 is to deliver RelA into the SCF^β-TrCP^ ubiquitin ligase. The SCF^β-TrCP^ ubiquitin ligase cannot target RelA independently of PDLIM2, because RelA does not contain the degron that can be recognized by β-TrCP. PDLIM2 has the ability to physically interact with both RelA and the SCF^β-TrCP^ ubiquitin ligase complex. Another important function is to stabilize ROC1 proteins, allowing the formation of the functional SCF^β-TrCP^ ubiquitin ligase for RelA ubiquitination and degradation.

## Association between PDLIM2 and the SCF^β-TrCP^ ubiquitin ligase complex

**Fig. 1:**
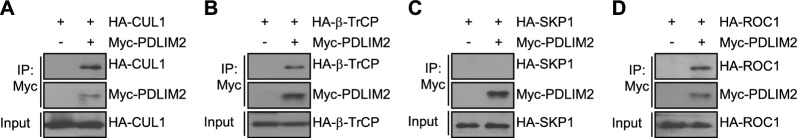
PDLIM2 physically interacts with the SCF^β-TrCP^ ubiquitin ligase complex in the cell nucleus. **A** Co-immunoprecipitation (IP) assays showing strong interaction between PDLIM2 and CUL1. **B** Co-IP assays showing strong association of PDLIM2 with β-TrCP. **C** Co-IP assays showing no association between PDLIM2 and SKP1. **D** Co-IP assays showing strong association between PDLIM2 and ROC1. Nuclear extracts of 293 cells expressing the indicated expression constructs were used for the Co-IP and immunoblotting (IB) assays. In D, nuclear extracts containing the same amount of ROC1 proteins were used for better comparison, because the ROC1 protein level was much higher in cells co-expressing PDLIM2 due to PDLIM2 stabilization of ROC1 (see the following Fig. 4). The same amounts of total nuclear extracts were used in A-C

To test whether PDLIM2, like ROC1 and many other RING finger proteins, functions in a complex to promote RelA ubiquitination and degradation, we first examined the association between PDLIM2 and CUL1 within cells, given the role of CUL1 in assembling the SCF^β-TrCP^ ubiquitin ligase complex and ROC1 binding in particular [9]. Since RelA ubiquitination and degradation by PDLIM2 happens in the nucleus and the SCF^β-TrCP^ ubiquitin ligase complex does exist in the nucleus [9], we used cell nuclear extracts for our assays. As shown in Fig. 1A, PDLIM2 directly bound to CUL1 when they were co-expressed in human embryonic kidney (HEK) 293 cells. Note, 293 cells do not express detectable endogenous PDLIM2 [7]. PDLIM2 also interacted with β-TrCP directly (Fig. 1B). However, PDLIM2 did not bind to SKP1 (Fig. 1C), which could be supported by the fact that SKP1 is an adaptor linking CUL1 to β-TrCP. Somewhat unexpectedly, PDLIM2 could physically associate with ROC1 as well (Fig. 1D). Nevertheless, these data suggested that PDLIM2 may form a larger functional complex with the prototypical SCF^β-TrCP^ ubiquitin ligase.

## Synergy of PDLIM2 with the SCF^β-TrCP^ ubiquitin ligase in promoting RelA ubiquitination

**Fig. 2:**
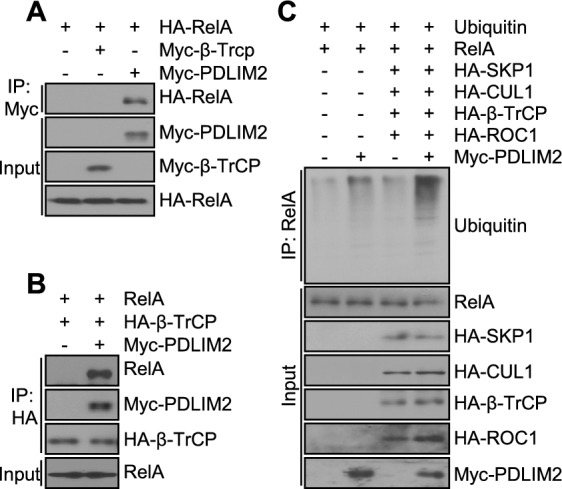
PDLIM2 synergizes with the SCF^β-TrCP^ ubiquitin ligase in promoting nuclear RelA ubiquitination. **A** Co-IP assays showing interaction of RelA with PDLIM2 but not β-TrCP. **B** Co-IP assays showing PDLIM2 recruitment of RelA to β-TrCP. **C** In vivo ubiquitination assays showing a strong synergy of PDLIM2 with the SCF^β-TrCP^ ubiquitin ligase in promoting RelA ubiquitination. Nuclear extracts containing the same amount of RelA proteins were used for better comparison

Given the intriguing data above, it is of interest to test the ability of the SCF^β-TrCP^ ubiquitin ligase in inducing RelA ubiquitination in the presence or absence of PDLIM2. To this end, we analyzed RelA protein sequence, because β-TrCP recognizes substrates through the doubly phosphorylated DSG motif (DpSGΦXpS, where Φ represents a hydrophobic and X represents any amino acid) [9]. Our computational analysis revealed no such degron motif in RelA. Indeed, unlike PDLIM2, β-TrCP failed to directly interact with RelA in 293 cells (Fig. 2A). However, a strong β-TrCP and RelA association were ready detected in the presence of PDLIM2 (Fig. 2B). Consistently, over-expression of the SCF^β-TrCP^ ubiquitin ligase failed to ubiquitinate RelA but could significantly increase RelA ubiquitination in the cells expressing PDLIM2 (Fig. 2C). These data suggested that PDLIM2 brings the SCF^β-TrCP^ ubiquitin ligase and RelA together, enhancing RelA ubiquitination.

## Indispensable role of ROC1 and β-TrCP in PDLIM2-mediated ubiquitination and degradation of RelA

**Fig. 3:**
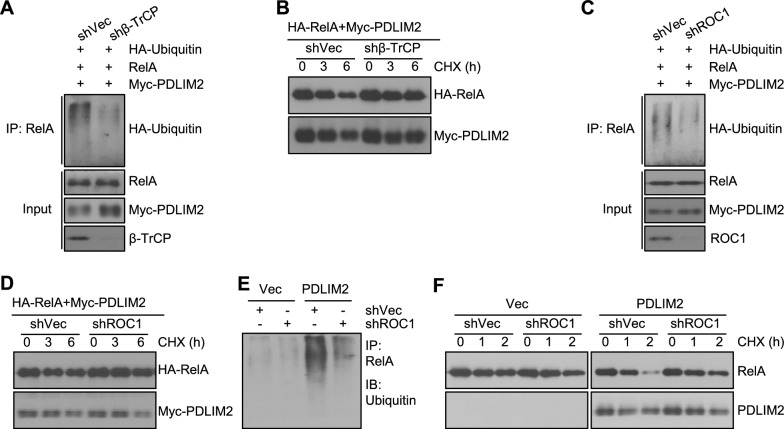
Silencing ROC1 or β-TrCP blocks PDLIM2-promoted ubiquitination and degradation of nuclear RelA. **A** In vivo ubiquitination assays showing prevention of PDLIM2-promoted RelA ubiquitination by β-TrCP shRNAs (shβ-TrCP) in 293 cells. **B** In vivo protein stability assays showing prevention of PDLIM2-induced RelA instability by β-TrCP shRNAs in 293 cells. **C** In vivo ubiquitination assays showing prevention of PDLIM2-promoted RelA ubiquitination by ROC1 shRNAs (shROC1) in 293 cells. **D** In vivo protein stability assays showing prevention of PDLIM2-induced RelA instability by ROC1 shRNAs in 293 cells. **E** In vivo ubiquitination assays showing prevention of PDLIM2-promoted RelA ubiquitination by ROC1 shRNAs in H460 cells. **F** In vivo protein stability assays showing prevention of PDLIM2-induced RelA instability by ROC1 shRNAs in H460 cells. Nuclear extracts containing the same amount of RelA proteins were used for the ubiquitination assays. For the protein stability assays, nuclear extracts containing the same amount of RelA proteins at the beginning of chasing were used

To determine if the SCF^β-TrCP^ ubiquitin ligase works downstream of PDLIM2 for RelA ubiquitination and proteasomal degradation, we tested the effect of β-TrCP specific short hairpin RNAs (shRNAs) on PDLIM2-promoted RelA ubiquitination and degradation in 293 cells. Indeed, β-TrCP shRNAs efficiently inhibited RelA ubiquitination by PDLIM2 and significantly increased RelA stability (Fig. 3A, B). Similarly, ROC1 shRNAs exhibited the same efficacy in blocking PDLIM2-promoted RelA ubiquitination and proteasomal degradation (Fig. 3C, D).

To further validate the studies, we examined the effect of ROC1 shRNAs on endogenous RelA. In this regard, we used the human lung cancer cell line H460, in which PDLIM2 is repressed but more importantly RelA is constitutively activated and expressed in the nucleus [2]. In the absence of exogenous PDLIM2, nuclear RelA was hardly ubiquitinated and exhibited a high stability, regardless of the expression of ROC1 shRNAs (Fig. 3E, F). Ectopic expression of PDLIM2 induced robust ubiquitination and rapid turnover of RelA, which were efficiently blocked by ROC1 shRNAs. Altogether, these data suggested that PDLIM2 promotes RelA ubiquitination and proteasomal degradation indirectly through the classical SCF^β-TrCP^ ubiquitin ligase.

## Stabilization of ROC1 by PDLIM2

**Fig. 4:**
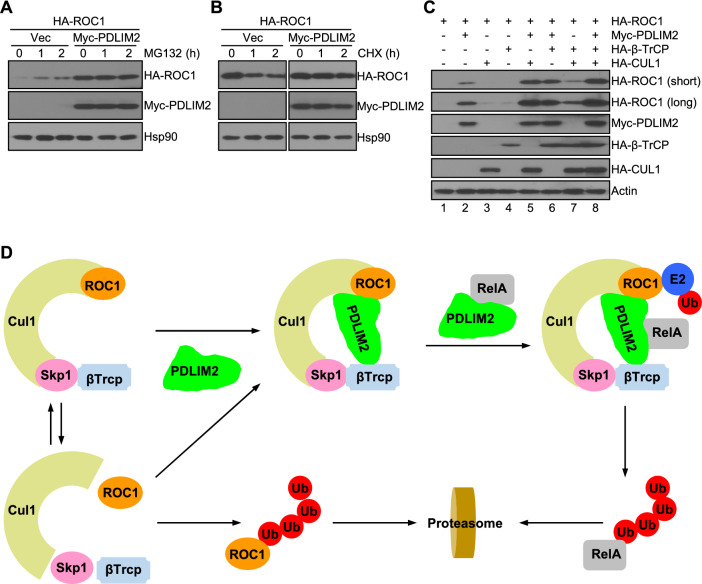
PDLIM2 stabilizes ROC1 and synergizes with CUL1 and β-TrCP for the maximal ROC1 stabilization. **A** IB assays showing MG132 accumulation of ROC1 proteins in the absence but not presence of PDLIM2 in 293 cells. **B** In vivo protein stability assays showing PDLIM2 stabilization of ROC1 proteins in 293 cells. Cell extracts containing the same amount of ROC1 proteins at the beginning of chasing were used. **C** IB assays showing the synergy of PDLIM2 with CUL1 and β-TrCP for the maximal ROC1 stabilization in 293 cells. **D** Modeling PDLIM2 as the E5 for RelA ubiquitination and proteasomal degradation

Notably, we observed a markedly higher level of ROC1 when PDLIM2 was co-expressed (Fig. 4A). Despite the significantly low expression when being expressed alone, ROC1 expression level was increased by the proteasome inhibitor MG132. On the other hand, the high level of ROC1 was not further increased by MG132 when it was co-expressed with PDLIM2. Consistently, PDLIM2 drastically increased ROC1 protein stability in the pulse-chase assays (Fig. 4B). These data suggested that PDLIM2 prevents ROC1 from its rapid proteasomal degradation.

CUL1 also stabilized ROC1, although at a much lower level compared to PDLIM2 (Fig. 4C, lane 3 vs. lanes 2 and 1). Interestingly, β-TrCP showed a similar effect in ROC1 stabilization as CUL1 (Fig. 4C, lane 4). The effect of β-TrCP would be indirectly through the endogenous CUL1 and SKP1 within the cells. Indeed, CUL1 and β-TrCP co-expression showed a synergy in stabilizing ROC1, to a level similar to that increased by PDLIM2 (Fig. 4C, lane 7 vs. lane 2). When CUL1 or β-TrCP was simultaneously expressed with PDLIM2, remarkably, ROC1 levels were further increased (Fig. 4C, lanes 5 and 6). The expression level of ROC1 reached to the highest when CUL1, β-TrCP and PDLIM2 all were expressed concomitantly (Fig. 4C, lane 8). These data suggested that in addition to recruiting RelA to the SCF^β-TrCP^ ubiquitin ligase, PDLIM2 plays an important role in stabilizing ROC1 and enhancing the formation of the functional SCF^β-TrCP^ ubiquitin ligase complex.

The PDLIM2/RelA axis has been linked to numerous pathogenic conditions and cancers in particular [2–8]. However, we do not know, until now, how PDLIM2 promotes nuclear RelA ubiquitination and proteasomal degradation, although PDLIM2 has been suggested to be a nuclear ubiquitin ligase [1]. The studies here show that PDLIM2 exerts the important function indirectly through the SCF^β-TrCP^ ubiquitin ligase. Besides this previously unidentified role, the SCF^β-TrCP^ ubiquitin ligase is well-known to ubiquitinate NF-κB inhibitors for degradation in the cytoplasm, freeing RelA and other NF-κB members to translocate to the nucleus and regulate gene transcription [7]. Thus, the SCF^β-TrCP^ ubiquitin ligase has two opposite roles in NF-κB regulation, ensuring a rapid but transient RelA activation in response to NF-κB stimuli. In the cytoplasm, it initiates NF-κB activation in response to NF-κB stimuli, and but in the nucleus, it turns off RelA activation, with the necessary help of PDLIM2.

Protein ubiquitination involves the sequential concerted action of E1, E2, and E3. This reaction starts with formation of a thiolester linkage between E1 and ubiquitin, followed by transfer of ubiquitin to an E2. Finally, E3 recruits a specific protein substrate to the E2-ubiquitin, where the ubiquitin is conjugated to a specific lysine in the protein substrate [9]. The serial actions of E1, E2, and E3 result in the poly-ubiquitination of the substrate. In certain cases, however, a ubiquitin-chain elongation factor named E4 is needed to bind to the oligo-ubiquitylated substrates for multi-ubiquitin chain assembly by E1, E2, and E3, yielding long ubiquitin chains [10].

PDLIM2 may represent a distinct and novel class of factors important for protein ubiquitination, acting as E3 enhancers, E5s (Fig. 4D). They recruit substrates that cannot be recognized by E3s, as indicated by the PDLIM2 recruitment of nuclear RelA to the SCF^β-TrCP^ ubiquitin ligase in the nucleus. They may also facilitate the formation and stabilization of E3s, especially the multi-subunit ones. In this regard, ROC1, the RING finger component of the SCF^β-TrCP^ ubiquitin ligase, is more stable within the E3 complex compared to being alone. However, PDLIM2 shows a much stronger ability in stabilizing ROC1 and synergizes with other components of the E3 for optimal ROC1 stabilization. The ROC1 stabilization function of PDLIM2 is dispensable for the ubiquitination and degradation of the targets of the SCF^β-TrCP^ ubiquitin ligase with β-TrCP degron sequence, such as the NF-κB inhibitor IκBα. It is highly plausible that binding to the target proteins further solidifies the SCF^β-TrCP^ complex, thereby preventing the dissociation from the complex and subsequent ubiquitination and degradation of ROC1.

In summary, the present studies demonstrate PDLIM2 as a novel E5 ubiquitin ligase enhancer that stabilizes ROC1 and recruits the ROC1-SCF^β-TrCP^ ubiquitin ligase to ubiquitinate and degrade nuclear RelA. They provide new mechanistic insights into how PDLIM2 serves as a common tumor suppressor and a critical immune regulator. They also expand our knowledge on the complex regulation and action of the ubiquitination and NF-κB pathways.

## Materials and methods

These are shown in the supplemental information.

### Supplementary Information

Supplementary Material 1.

## Data Availability

All data generated or analyzed during this study are included in this published article and its supplementary information files.
