# Supplementary material for: PDLIM2 is a novel E5 ubiquitin ligase enhancer that stabilizes ROC1 and recruits the ROC1-SCF ubiquitin ligase to ubiquitinate and degrade NF-κB RelA
Source: Cell Biosci. 2024 Jul 30;14:99. doi: 10.1186/s13578-024-01281-x (PMC11287889; doi:10.1186/s13578-024-01281-x)
Supplement: Supplementary file 1 — Supplementary Material 1. [file 13578_2024_1281_MOESM1_ESM.docx]

**Additional information**

**Additional file 1: Materials and Methods**

**Expression vectors and reagents**

Expression vectors encoding RelA, ubiquitin, HA-tagged RelA, HA-tagged β-TrCP, HA-tagged ubiquitin and myc-tagged PDLIM2 were described before [6]. HA-tagged CUL1, HA-tagged ROC1 and HA-tagged SKP1 were cloned into the expression vectors pcDNA or pCMV4. Lentiviral vector pLL3.7 used to express shRNAs specifically against human β-TrCP or ROC1 were described previously [2, 6]. PDLIM2 and RelA antibodies were described previously [5, 6]. HA antibody (12CA5) and HRP-conjugated HA antibody (3F10) were from Roche Molecular Biochemicals (Indianapolis, IN). Myc antibody (9E10), ubiquitin antibody (P4D1), were from Santa Cruz Biotechnology (Santa Cruz, CA). The proteasome inhibitor MG132, and protein synthesis inhibitor cycloheximide (CHX) were from Biomol (Plymouth Meeting, PA).

**Cell culture and transfection**

HEK293 and H460 cells were cultured in Dulbecco modified Eagle medium (DMEM) supplemented with 10% fetal bovine serum (FBS) and 2 mM l-glutamine and transfected using Lipofectamine 2000 (Invitrogen, Frederick, MD) in accordance with the manufacturer’s instructions. Twenty-four hours after the transfection, cell nuclear extracts were prepared and subjected to IB, IP, *in vivo* ubiquitin conjugation and/or *in vivo* protein stability assays [2, 5, 6].

**shRNA-mediated gene silencing**

293 and H460 cells lines stably expressing shRNAs β-TrCP or ROC1 by pLL3.7 were generated as described before [2, 5, 6]. The sequences for shRNA are: for β-TrCP, GTGGAATTTGTGGAACATC; ROC1, TGCCATCTGCAGGAACCACAT. These shRNAs were synthesized by Dharmacon Research, Inc. (Lafayette, CO).

**Nuclear subcellular fractionation, IB, and IP assays**

The indicated cells were lysed in the hypotonic buffer (20 mM HEPES, pH 8.0, 10 mM KCl, 1 mM MgCl_2_, 0.1% [vol/vol] Triton X-100, and 20% [vol/vol] glycerol) and swelled on ice for 15 min, followed by centrifugation for 5 min at 4 °C and 12,000 × *g*. The supernatant was cytoplasmic extract. The pellet was further washed three times with the hypotonic buffer, suspended in insoluble nuclear buffer (20 mM Tris, pH 8.0, 150 mM NaCl, 1% [wt/vol] SDS, 1% [vol/vol] NP-40, and 10 mM iodoacetamide), and rotated at 4 °C for 15 min, followed by centrifugation for 5 min at 4 °C and 12,000 × *g*. The resultant supernatant was nuclear extract [2, 6]. Whole cell extracts were made using radioimmunoprecipitation assay (RIPA) buffer (50 mm Tris-HCl, pH 7.4, 150 mm NaCl, 1 mm EDTA, 0.25% sodium deoxycholate, 1% Nonidet P-40, and 1 mm dithiothreitol). All the lysis buffers were supplemented with 1 mM phenylmethylsulfony fluoride (PMSF) and a protease inhibitor cocktail (Roche Molecular Biochemicals). The cell extracts were used for IP and/or IB assays as described before [2, 6].

***In vivo* ubiquitin conjugation assay**

293 or H460 cells transfected with the indicated constructs with or without exogenous ubiquitin or HA-tagged ubiquitin were treated with MG132 for 2 hours before nuclear extraction for IP using anti-RelA. The ubiquitin-conjugated RelA pulled down by IP was detected by IB using anti-ubiquitin or anti-HA-HRP [6].

***In vivo* protein stability assay**

Cells were treated with 10 μM CHX, followed by chase for the indicated time-period in the presence or absence of MG132, and IB to detect the indicated proteins [6].
